# AI-Assisted WES Variant Interpretation in Neurodevelopmental Disorders: A Narrative Review of Existing Tools

**DOI:** 10.3390/genes17101276

**Published:** 2026-10-14

**Authors:** Simone Treccarichi, Mirella Vinci, Antonino Musumeci, Rosanna Galati Rando, Antonio Fallea, Desiree Brancato, Francesca Bruno, Concetta Federico, Salvatore Saccone, Maurizio Elia, Francesco Cali

**Affiliations:** 1 Oasi Research Institute-IRCCS, 94018 Troina, Italy; streccarichi@oasi.en.it (S.T.); mvinci@oasi.en.it (M.V.); amusumeci@oasi.en.it (A.M.); rgalati@oasi.en.it (R.G.R.); afallea@oasi.en.it (A.F.); melia@oasi.en.it (M.E.); 2 Department of Biological, Geological and Environmental Sciences, University of Catania, 95124 Catania, Italy; desiree.brancato@phd.unict.it (D.B.); concetta.federico@unict.it (C.F.); salvatore.saccone@unict.it (S.S.); 3 Department Medicine and Surgery, Kore University of Enna, 94100 Enna, Italy; francesca.bruno@unikore.it

**Keywords:** neurodevelopmental disorders, artificial intelligence, whole-exome sequencing, variant interpretation, variant prioritization, pathogenicity prediction, protein structure prediction

## Abstract

**Background/Objectives**: Neurodevelopmental disorders (NDDs) comprise a heterogeneous group of conditions characterized by alterations in brain development and function, frequently associated with complex genetic architectures. The widespread implementation of next-generation sequencing, particularly whole-exome sequencing (WES), has substantially increased the identification of potentially disease-associated variants, shifting the major challenge from variant detection to their interpretation and prioritization. This review examines the emerging contribution of artificial intelligence (AI) to WES variant interpretation in NDDs, with particular emphasis on AI-assisted variant prioritization and protein structural modelling. **Methods**: We reviewed conventional computational and emerging AI-driven approaches applicable to different stages of WES interpretation, including variant pathogenicity and functional-effect prediction, evolutionary conservation, splicing assessment, phenotype-informed prioritization, and protein structure prediction. Their methodological principles, applications, and major limitations were critically examined within the context of neurodevelopmental genomics. **Results**: AI and machine-learning approaches increasingly integrate genomic, phenotypic, functional, and structural information, supporting phenotype-aware candidate prioritization and molecular contextualization of selected variants. However, computational outputs are affected by model-specific limitations and should not be considered independent evidence of pathogenicity. Their added value depends primarily on the complementarity and biological relevance of the information provided, rather than on predictor concordance alone. **Conclusions**: This narrative review highlights that AI-assisted WES interpretation should prioritize complementary, mechanism-relevant evidence over computational concordance. AI-assisted tools should therefore be selected according to variant class and disease mechanism and integrated with independent clinical, genetic, and functional evidence.

## 1. Introduction

Neurodevelopmental disorders (NDDs) comprise a clinically heterogeneous group of conditions arising from alterations in brain development and affecting cognitive, behavioral, motor, and language functions. According to the Diagnostic and Statistical Manual of Mental Disorders, Fifth Edition (DSM-5), NDDs encompass several conditions, including intellectual disability (ID), autism spectrum disorder (ASD), attention-deficit/hyperactivity disorder (ADHD), communication disorders, specific learning disorders, and motor disorders [1,2]. NDDs represent a substantial global health burden because of their relatively high prevalence, frequent lifelong course, and considerable impact on affected individuals, families, healthcare systems, and society. Despite major advances in their clinical characterization, establishing an accurate etiological diagnosis remains challenging, particularly because of the extensive phenotypic overlap and frequent occurrence of comorbid neurological and psychiatric manifestations. Genetic factors play a pivotal role in the etiology of NDDs, although their genetic architecture is remarkably complex [3,4,5]. Disease-associated alterations range from single-nucleotide variants (SNVs) and small insertions/deletions (indels) to copy-number variants (CNVs) and larger structural rearrangements. Moreover, NDDs are characterized by extensive genetic heterogeneity, with different variants within the same gene potentially resulting in distinct phenotypes, and alterations in different genes converging toward overlapping clinical manifestations [6,7]. The widespread implementation of NGS, particularly whole-exome sequencing (WES), has substantially increased the ability to investigate this genetic complexity and identify candidate disease-associated variants. WES is often used as a first-tier genetic test for diagnostic purposes [8]. Nevertheless, each exome contains thousands of genomic variants, only a small fraction of which may be relevant to the patient’s phenotype. Consequently, the interpretation and prioritization of variants, rather than their identification alone, have become major challenges in neurogenomic diagnostics. Conventional variant interpretation relies on the integration of multiple sources of evidence, including population allele frequency, inheritance pattern, evolutionary conservation, predicted functional consequences, segregation data, phenotype–genotype associations, and information available in clinical and population databases [9,10]. Although these approaches remain fundamental, the increasing amount and complexity of genomic data have highlighted the limitations of manual interpretation and of individual rule-based or conventional computational predictors [11]. In this context, machine-learning and AI approaches have progressively emerged as powerful tools for genomic interpretation. AI-based models can integrate complex and multidimensional biological information to estimate variant pathogenicity, predict alterations in splicing or protein function, and facilitate the prioritization of potentially disease-associated variants [12,13]. AI-based approaches are increasingly being applied across different areas of genetics, ranging from clinical genomics to forensic genetics [14]. More recently, advances in deep learning have extended these applications to protein structural biology, with models such as AlphaFold, RoseTTAFold, and ESMFold providing new opportunities to investigate the structural context of candidate variants and their potential consequences on protein conformation and molecular interactions [15]. Within this rapidly evolving landscape, the integration of computational variant prioritization with AI-driven structural modelling may provide a valuable framework for moving from genomic data to biological interpretation.

This review critically examines the principal computational and AI-based approaches applicable to the interpretation of variants identified through WES in NDDs, with particular emphasis on pathogenicity prediction, splicing assessment, phenotype-informed prioritization, and protein structural modelling. Unlike recent reviews broadly addressing AI and deep learning in genomic analysis [12,13,16], we adopt a WES-centered, workflow-oriented perspective. Beyond describing individual tools, we examine when different computational approaches provide complementary information, when their outputs may be partially redundant, and how their interpretive value changes according to variant class and expected molecular mechanism. Particular attention is given to the distinction between computational prediction, candidate prioritization, and formal clinical variant classification.

### Searching Strategy

This narrative review was based on a literature search conducted using Google Scholar, PubMed, and Scopus. The search focused on studies addressing the application of AI, machine learning, and deep-learning approaches to genomic variant interpretation in NDDs, with particular emphasis on WES, variant pathogenicity prediction, splicing prediction, phenotype-driven variant and gene prioritization, sequence-to-function modelling, and protein structure prediction. Search terms included combinations of “artificial intelligence”, “machine learning”, “deep learning”, “neurodevelopmental disorders”, “whole-exome sequencing”, “variant interpretation”, “variant prioritization”, “pathogenicity prediction”, “splicing prediction”, “phenotype prioritization”, “protein structure prediction”, “AlphaFold”, and related terms. The original literature search included peer-reviewed original research articles and reviews published in English up to July 2026. During manuscript revision, supplementary targeted searches were performed to incorporate relevant methodological developments published or made publicly available up to 31 August 2026. Given the rapidly evolving nature of AI applications in genomics, selected recent preprints were additionally considered when they described emerging methodological approaches directly relevant to the scope of this review and for which a peer-reviewed publication was not yet available. Preprints were evaluated separately from peer-reviewed evidence and are explicitly identified as such throughout the manuscript. Their inclusion was intended to capture recent methodological developments rather than to support established clinical conclusions. Seminal studies describing the development and validation of major computational tools were also included irrespective of publication date. Studies were selected on the basis of their relevance to the scope of this narrative review rather than through a formal systematic-review protocol. The tools included in Tables were selected as representative examples based on their methodological relevance to variant interpretation or functional contextualization, availability of a primary methodological description, and relevance to the scope of this review. These tables are intended to provide representative comparisons rather than exhaustive catalogues of available tools. When available, original publications describing tool development were prioritized, together with independent validation or benchmarking studies relevant to their performance and limitations.

Titles, abstracts, and relevant full texts were screened by S.T. and M.V., and disagreements regarding inclusion were resolved by S.S. and F.C. When necessary to describe software availability, implementation, score interpretation, or current functionality, official software documentation, databases, repositories, and web-based resources were consulted. Commercial platforms were considered only when directly relevant to the implementation or accessibility of the computational approaches discussed and were not used as primary sources of evidence regarding model performance.

## 2. AI Throughout the Neurogenomics Workflow

### 2.1. Conventional WES Data Analysis Workflow

WES has become a widely adopted approach for investigating the genetic basis of NDDs, enabling the simultaneous analysis of the protein-coding regions of thousands of genes. The analytical workflow following WES can generally be divided into primary, secondary, and tertiary analysis [17,18]. Primary analysis involves the conversion of raw sequencing signals into nucleotide sequences and the generation of sequencing reads, typically stored in FASTQ files, together with the corresponding base-quality scores [19]. Quality control represents an essential step at this stage to identify potential technical issues, including low-quality reads, sequencing artifacts, adapter contamination, and insufficient sequencing depth.

Secondary analysis converts sequencing reads into genomic variant information. High-quality reads are aligned against a human reference genome, followed by post-alignment processing and variant calling [20]. SNVs and small insertions and deletions (indels) are subsequently identified and stored in Variant Call Format (VCF) files. Depending on the analytical pipeline, WES data can also be exploited for the detection of CNVs, although their identification from exome sequencing presents additional technical challenges compared with SNV and indel detection. More generally, although WES is particularly effective for the identification of coding SNVs and small indels, it does not comprehensively interrogate all classes of genomic variation. Non-coding variants, complex structural rearrangements, and some copy-number alterations may require complementary approaches, including whole-genome sequencing and cytogenomic analyses. Modern cytogenomics extends diagnostic resolution to a broad spectrum of balanced and unbalanced structural variants and therefore remains complementary to sequence-based approaches in the investigation of genetically heterogeneous disorders [21]. Quality metrics and variant-level filters are then applied to minimize sequencing and calling artifacts and retain high-confidence genomic variants.

Tertiary analysis represents the transition from variant detection to biological and clinical interpretation [22]. Identified variants are annotated using information derived from population, clinical, evolutionary, and functional databases [23]. Common variants are generally deprioritized according to population allele frequencies, while the predicted molecular consequence, affected transcript, inheritance model, disease–gene associations, segregation data, and compatibility with the patient’s phenotype are progressively considered. Evolutionary conservation and computational predictions of functional impact provide additional evidence for assessing candidate variants. This multistep filtering process progressively reduces the large number of variants detected in each individual to a restricted set of candidates requiring detailed interpretation and, when appropriate, classification according to established clinical guidelines.

Despite continuous improvements in sequencing and bioinformatic pipelines, tertiary analysis remains one of the major bottlenecks of WES-based diagnostics. The interpretation of rare variants is particularly challenging in NDDs because of extensive genetic and phenotypic heterogeneity, variable expressivity, incomplete penetrance, and the large number of genes potentially contributing to overlapping clinical presentations [24,25]. Moreover, different computational predictors may provide conflicting results, while variants in poorly characterized genes or variants of uncertain significance (VUS) frequently lack sufficient evidence for definitive classification [25]. Consequently, conventional filtering and manual curation can be time-consuming and strongly dependent on available knowledge, highlighting the need for approaches capable of integrating increasingly complex genomic and phenotypic information. Recent WES-based studies in NDDs illustrate this interpretative challenge, particularly when rare variants are identified in genes with limited or emerging disease associations. In such cases, the integration of genomic and phenotypic information can support the prioritization of candidate variants and genes [26,27,28]. Studies involving genes such as *ARIH2*, *PSMC5*, *USP24*, and *RAPGEF6* have required the integration of inheritance pattern, population frequency, gene function, phenotype compatibility, computational predictions, and available experimental and literature evidence to assess the potential contribution of individual variants and genes to neurodevelopmental phenotypes [29,30,31]. These interpretative challenges are further amplified by features that are particularly relevant to NDDs. A substantial proportion of severe neurodevelopmental phenotypes is associated with de novo variation, while incomplete penetrance and variable expressivity may complicate the interpretation of inherited variants and genotype–phenotype correlations. Post-zygotic mosaicism represents an additional challenge, as disease-associated variants may be present at low allelic fractions, and their detection can depend on sequencing depth and the tissue analyzed [32,33]. Moreover, neurodevelopmental phenotypes evolve over time, and the clinical presentation of young children may be incomplete because specific cognitive, behavioral, neurological, or dysmorphic features become apparent only at later developmental stages [34,35]. Consequently, phenotype-driven computational prioritization performed early in life may be affected by incomplete clinical information and may require periodic re-evaluation as the phenotype evolves. Finally, the coexistence of multiple molecular diagnoses or variants contributing to distinct components of the phenotype may result in blended presentations that cannot always be adequately represented by single-gene prioritization models.

### 2.2. Integration of Artificial Intelligence into WES Interpretation and Variant Prioritization

Both machine and deep learning are increasingly being integrated into genomic analysis to support the interpretation and prioritization of variants identified through WES [13]. Although AI-based approaches can potentially contribute to different stages of the sequencing workflow, their impact is particularly relevant during tertiary analysis, where heterogeneous sources of biological and clinical evidence must be combined to distinguish potentially disease-associated variants from the large background of genomic variation. In fact, conventional variant filtering frequently relies on predefined thresholds and independent criteria, such as allele frequency, predicted molecular consequence, evolutionary conservation, or previously established disease associations [36,37].

By contrast, AI-based models provide a complementary approach by learning complex relationships from large genomic and biological datasets and integrating multiple features into quantitative predictions [38,39]. Depending on their architecture and training strategy, these models can incorporate information related to sequence context, amino acid properties, evolutionary constraint, protein domains, regulatory features, splicing patterns, and other molecular characteristics. This approach has enabled the development of increasingly sophisticated predictors designed to estimate the probability that a genomic alteration may disrupt normal molecular function or contribute to disease. Machine-learning approaches therefore play an increasingly important role in variant prioritization [38,39]. Rather than considering individual annotations in isolation, computational frameworks can combine multiple lines of evidence to rank candidate variants according to their predicted biological or clinical relevance. Deep learning has further expanded these capabilities by enabling models to extract complex sequence features directly from large-scale genomic datasets, reducing the dependence on manually defined features. More recently, advances in protein language models and other foundation-model approaches have extended AI-assisted interpretation from genomic sequence to amino acid sequence and protein-level information, providing additional opportunities for evaluating missense variation [40]. AI-driven pathogenicity prediction tools, as well as protein structure prediction and modelling approaches, will be discussed in greater detail in the following sections.

The potential contribution of AI becomes particularly relevant when genomic information is integrated with phenotypic data. In NDDs, where substantial phenotypic overlap exists across genetically distinct conditions, phenotype-driven prioritization can help identify variants affecting genes whose known or predicted functions are compatible with the clinical presentation. Computational approaches incorporating standardized phenotype descriptions, particularly Human Phenotype Ontology (HPO) terms, can rank candidate genes and variants according to genotype–phenotype similarity [41]. The combination of genomic, functional, and phenotypic evidence therefore represents an important step toward more comprehensive AI-assisted prioritization strategies.

Importantly, AI-generated predictions should not be regarded as independent evidence of pathogenicity or as substitutes for clinical interpretation and experimental validation. Their performance depends on the quality and composition of training datasets, and predictions may be influenced by biases toward well-characterized genes, variant classes, populations, and disease mechanisms. Computational scores should therefore be interpreted together with population frequency, segregation, inheritance pattern, phenotype compatibility, functional evidence, and established variant-classification frameworks. Within this context, AI should be considered an additional layer of evidence capable of supporting, rather than replacing, expert genomic interpretation. This aspect has recently received increasing attention, particularly in the context of genetic counselling [42,43,44] and will be further discussed in a subsequent section of this review.

**Figure 1:**
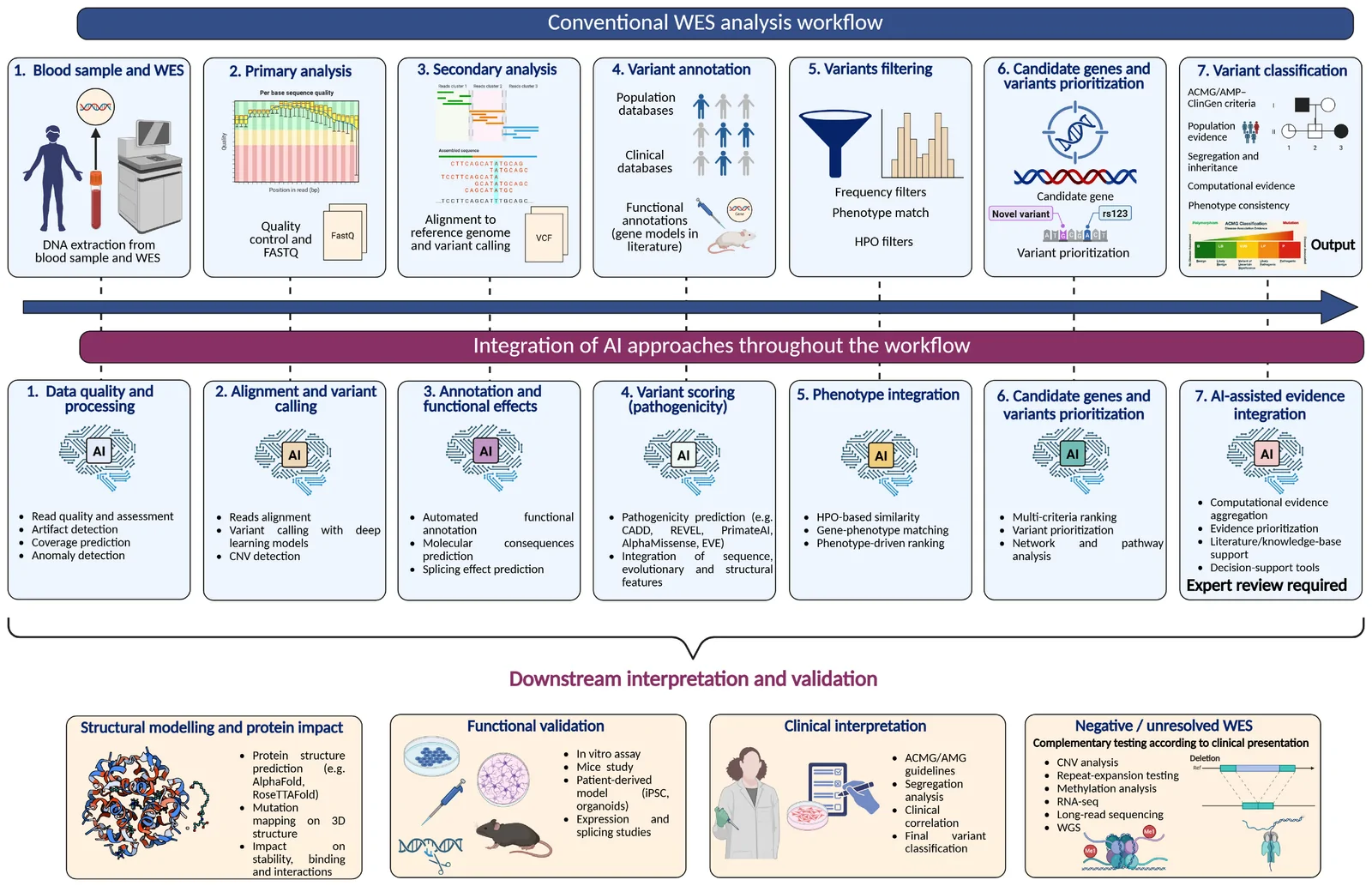
Integration of artificial intelligence approaches throughout the whole-exome sequencing workflow. The upper panel summarizes the conventional WES analytical workflow, from sample processing and sequencing to variant annotation, filtering, candidate variant prioritization, and classification according to ACMG/AMP–ClinGen criteria. The middle panel illustrates potential applications of AI-based approaches across different analytical stages, including data quality assessment, alignment and variant calling, functional annotation, pathogenicity prediction, phenotype integration, and candidate gene and variant prioritization. Following prioritization and classification, selected variants may undergo downstream investigation through AI-assisted protein structural modelling, experimental functional validation, and clinical interpretation according to established variant-classification frameworks. AI-based approaches should be considered complementary tools supporting, rather than replacing, expert genomic and clinical interpretation, integrating genomic, segregation, phenotypic, and available functional evidence. In negative or unresolved WES cases, complementary investigations, including CNV analysis, repeat-expansion testing, methylation analysis, RNA sequencing, long-read sequencing, or whole-genome sequencing, may be considered according to the clinical presentation.

The integration of AI into tertiary analysis thus represents a transition from predominantly rule-based variant filtering toward multidimensional and data-driven prioritization [22,45]. Following the initial reduction in variants through conventional quality and frequency filters, computational pathogenicity and functional-effect predictors—including conventional, machine-learning, and deep-learning approaches—can provide additional evidence for ranking candidate variants. The highest-priority candidates can subsequently undergo more detailed molecular investigation, including assessment of splicing consequences and protein structural modelling. This progressive workflow establishes a conceptual continuum from WES data generation to AI-assisted variant prioritization and, ultimately, to the structural and biological interpretation of candidate disease-associated variants. The conventional WES analytical workflow and the potential integration of AI-based approaches across its different stages are schematically illustrated in Figure 1, highlighting their contribution from sequence data processing to variant interpretation, prioritization, and downstream functional characterization.

## 3. AI in Variant Interpretation and In Silico Tools for Pathogenicity Prediction

### 3.1. From Conventional Computational Predictors to AI-Driven Approaches

In silico prediction has become an integral component of variant interpretation, providing computational estimates of the potential functional consequences of genomic alterations [11,46,47]. Historically, pathogenicity prediction relied primarily on evolutionary conservation, physicochemical differences between amino acids, protein sequence properties, and predefined biological features. The increasing availability of large genomic datasets and experimentally characterized variants has progressively enabled the development of more sophisticated approaches based on machine learning (ML), deep learning (DL), and generative modelling [13]. These methods can integrate multiple and potentially nonlinear features, providing increasingly comprehensive estimates of variant deleteriousness or pathogenicity.

Computational predictors differ substantially in their methodological foundations [48]. Supervised machine-learning algorithms are trained using variants with predefined labels, generally representing pathogenic/deleterious and benign/neutral classes, and learn combinations of features capable of discriminating between them. Ensemble approaches further combine information derived from multiple individual predictors or genomic annotations into a unified score. Conversely, unsupervised or semi-supervised approaches can infer patterns of biological constraint without relying exclusively on clinically assigned pathogenicity labels. More recently, deep learning architectures have enabled models to learn complex representations directly from genomic or protein sequences and large-scale evolutionary information. Understanding these methodological differences is essential because the training strategy, input features, and reference datasets of each predictor directly influence its performance, applicability, and potential biases.

### 3.2. Pathogenicity Prediction of Coding Variants

Combined Annotation Dependent Depletion (CADD) represents one of the most widely used frameworks for estimating variant deleteriousness [49]. CADD integrates numerous genomic annotations into a single score by contrasting variants observed in the human population with simulated variants. Its broad applicability enables the assessment of different classes of genomic variation, extending beyond protein-coding substitutions. Importantly, CADD scores should be interpreted as estimates of variant deleteriousness rather than direct probabilities of clinical pathogenicity. Recent versions of CADD have further evolved through the implementation of machine-learning-based approaches for variant scoring and deleteriousness prediction [50].

Rare Exome Variant Ensemble Learner (REVEL) was specifically developed for the prediction of rare missense variant pathogenicity [51]. REVEL uses an ensemble machine-learning strategy integrating multiple individual pathogenicity and conservation scores to generate a unified prediction. Its specific optimization for rare missense variants has contributed to its widespread implementation in clinical variant interpretation. However, because several underlying predictors share related biological features, the resulting score should not be considered completely independent of other computational evidence derived from conservation and functional annotations.

PrimateAI and its subsequent developments introduced deep learning into missense variant interpretation by leveraging protein sequence information together with evolutionary data derived from primate populations [52]. The underlying rationale is that naturally occurring variation across closely related species and populations can provide information regarding tolerance to amino acid substitutions. These approaches illustrate the transition from manually engineered combinations of annotations toward models capable of learning complex representations of protein variation from large biological datasets.

AlphaMissense represents a further development in deep-learning-based missense variant prediction [53]. Built upon concepts derived from AlphaFold and protein sequence modelling [54,55,56], AlphaMissense integrates evolutionary and structural-context information to estimate the likelihood that amino acid substitutions are pathogenic. Its application at the proteome scale has enabled pathogenicity predictions for a substantial proportion of theoretically possible human missense variants, including variants that have not yet been observed clinically. Nevertheless, AlphaMissense predictions represent computational evidence and should not be interpreted as direct demonstrations of pathogenic mechanisms.

The Evolutionary Model of Variant Effect (EVE) adopts a conceptually different strategy [57]. Rather than being trained primarily on clinically labelled pathogenic and benign variants, EVE uses generative models of evolutionary sequence variation to estimate whether a given amino acid substitution is compatible with the evolutionary constraints acting on a protein family [58]. Variants that strongly deviate from the learned distribution of evolutionarily tolerated sequences can consequently be prioritized as potentially deleterious. This approach reduces dependence on existing clinical labels and illustrates the potential of unsupervised or generative AI models for variant interpretation, particularly for variants with limited previous clinical evidence. EVE generates a continuous pathogenicity score ranging from 0 to 1, with higher values indicating a greater predicted propensity toward pathogenicity. In addition, the model provides variant class assignments and estimates of classification uncertainty. An important limitation of evolution-based approaches is their dependence on the depth and quality of the multiple sequence alignment (MSA), which may reduce prediction reliability for poorly conserved or insufficiently represented protein families. This issue may be particularly relevant for emerging candidate genes with limited evolutionary information. More recent evolution-based frameworks, such as ProteoCast, explicitly incorporate MSA-derived confidence metrics at the protein and residue levels, allowing prediction scores to be interpreted together with the amount of evolutionary information supporting them [59]. Thus, for evolution-based variant-effect prediction, the predicted score should be considered together with the corresponding confidence and alignment coverage rather than interpreted in isolation.

Given the focus of this review on AI-assisted genomic interpretation, the following tools were selected as representative pathogenicity predictors incorporating machine-learning or deep-learning approaches. Specifically, CADD, REVEL, PrimateAI, PrimateAI-3D, EVE, and AlphaMissense were included to illustrate different computational strategies, ranging from ensemble and supervised learning to deep neural networks, unsupervised generative modelling, and structure-informed approaches. This selection was intended to provide representative examples of the methodological evolution of ML-based variant-effect prediction rather than an exhaustive comparison of available predictors. In fact, these tools demonstrate that pathogenicity prediction is progressively moving from individual conservation- or feature-based algorithms toward models capable of integrating multidimensional genomic, evolutionary, and structural information. However, their scores are not directly interchangeable. Each model addresses a specific prediction task and is influenced by its training data, architecture, input features, and definition of pathogenicity or deleteriousness. The combined interpretation of multiple computational outputs therefore requires careful consideration of methodological overlap and potential circularity. Based on the evidence discussed in this review, we emphasize that, despite the increasing implementation of automated approaches for variant classification and prioritization, human expertise remains essential for the critical evaluation of computational evidence and clinical decision-making. This is particularly relevant in genetic counselling, where qualified genetics professionals play a crucial role in contextualizing genomic findings and communicating their clinical implications to patients and families.

The principal characteristics of the pathogenicity predictors discussed in this review are summarized in Table 1, including their computational framework, main input features, training strategy, and the interpretation of their output scores.

Direct comparison of these predictors requires caution, as their performance may vary according to variant class, gene, disease mechanism, and benchmarking dataset. Supervised and ensemble models may be affected by circularity between training and evaluation datasets, while evolutionary or structure-informed approaches may have limitations in poorly conserved or intrinsically disordered regions. Training data may also introduce ancestry-related and ascertainment biases. Importantly, independent benchmarking studies have confirmed substantial gene- and context-dependent variability, indicating that no single predictor can be considered universally optimal, even when recent models such as AlphaMissense show high overall performance [61,62].

In the context of NDDs, these tools are particularly relevant because of the extensive locus and allelic heterogeneity characterizing this group of disorders, in which rare de novo or inherited coding variants frequently require prioritization among multiple plausible candidates [63]. Computational variant-effect prediction may therefore help reduce the interpretative burden associated with WES, especially when several variants of uncertain significance are identified in genes potentially related to neurodevelopment. In NDDs, these tools are particularly relevant because extensive locus and allelic heterogeneity coexists with a substantial contribution of rare de novo and inherited coding variants affecting functionally diverse genes, including ion channels, synaptic proteins, transcriptional regulators, and chromatin-remodelling factors.

Computational predictions should be distinguished from formal clinical variant classification. Within the ACMG/AMP framework, computational evidence may contribute to PP3 or BP4 when supporting a deleterious or benign effect, respectively [64]. ClinGen recommendations further emphasize the use of calibrated, tool-specific thresholds and caution against treating concordant predictions as independent evidence, as different tools may share annotations, training data, or underlying predictors [65]. Accordingly, computational scores should be integrated with independent clinical, genetic, and functional evidence rather than directly translated into pathogenicity classifications. Deleteriousness or functional-effect prediction, variant prioritization, and clinical variant classification therefore represent distinct, although complementary, levels of genomic interpretation.

The performance of variant-effect predictors is strongly influenced by benchmark composition. In a comparison of 23 computational methods, Li et al. (2018) [66] reported an AUC of 0.920 for REVEL, with 81.12% sensitivity and 87.07% specificity in a ClinVar missense-variant benchmark. By contrast, Gunning et al. (2021) [67] observed generally lower performance in a clinically representative dataset derived from diagnostic and research sequencing, with REVEL achieving the highest AUC (0.818). Benchmarking may also be affected by data circularity when variants or genes represented during model development overlap with evaluation datasets, potentially producing overly optimistic performance estimates [68]. These findings indicate that predictor performance is dataset-dependent and should not be assumed to generalize across different genes, variant classes, or clinical settings.

The interpretation of computational predictions should therefore consider independently benchmarked performance rather than the simple number of concordant scores. Agreement between variant-effect predictors and multiplexed functional assays has been associated with performance in clinical variant classification, whereas discrepancies may reflect specific molecular features and disease mechanisms rather than random error [69]. Moreover, predictor performance may be gene- and disease-dependent, and disease-specific calibration can alter the relative performance of individual tools [70]. Predictor interpretation should therefore consider the gene, disease mechanism, variant context, and available independent functional evidence.

To illustrate the integration of computational predictions with clinical and genetic evidence, we selected two missense variants in *SCN2A* showing different computational prediction patterns. The c.643G>A, p.(Ala215Thr) variant (rs1131691927) corresponds to NM_001040143.1:c.643G>A and NC_000002.12:g.165309202G>A (GRCh38) and was reported as a heterozygous de novo variant in a patient with severe neonatal-onset epilepsy. According to the OMIM database, SCN2A is associated with developmental and epileptic encephalopathy (MIM #613721). The p.(Ala215Thr) variant is reported in ClinVar (Variation ID: 851358; accession: VCV000851358.11), while no population frequency was reported in gnomAD v4.1. As a contrasting example, the SCN2A NM_001040142.2:c.1399G>A, p.(Ala467Thr) variant (rs745774658) was selected because it shows discordant computational predictions and is present at a low allele frequency in gnomAD v4.1 (AF = 1.859 × 10^−5^). Table 2 compares the computational predictions obtained for these two variants using CADD, REVEL, and AlphaMissense (resources accessed on 17 August 2026).

The p.(Ala215Thr) variant represents an informative NDD example because it affects the developmentally regulated neonatal 6N transcript of *SCN2A* and was identified de novo in a patient with severe neonatal-onset epilepsy following transcript-level re-evaluation. CADD, REVEL, and AlphaMissense were selected as representative predictors to illustrate how computational predictions may converge or diverge even within the same gene. While all three predictors support a deleterious effect for p.(Ala215Thr), p.(Ala467Thr) shows discordant predictions, with a relatively elevated CADD score (23.5) but benign-supporting REVEL (0.269) and AlphaMissense (0.08) scores. The latter variant was classified as benign by the ClinGen Epilepsy Sodium Channel Variant Curation Expert Panel. More broadly, *SCN2A* variants should be interpreted within gene-specific ACMG/AMP specifications, which integrate calibrated population, computational, and functional evidence, including electrophysiological data [71]. Thus, computational predictions should be considered complementary evidence rather than directly translated into clinical classification. These variants are presented as illustrative examples of concordant and discordant computational outputs and are not intended to constitute an independent validation of predictor performance or of the conceptual workflow discussed in this review.

### 3.3. Computational and AI-Driven Prediction of Splicing Alterations

The pathogenic consequences of genomic variants are not restricted to amino acid substitutions. Variants affecting canonical splice sites, nearby intronic regions, exonic regulatory elements, or deeper intronic sequences may disrupt pre-mRNA processing and contribute to disease [72]. Conventional splice-site predictors have traditionally relied on sequence motifs and predefined statistical models; however, deep learning has substantially expanded the ability to identify potentially splice-altering variants.

SpliceAI is a deep neural network developed to predict splice junctions directly from primary nucleotide sequence and to estimate the probability that a genomic variant induces splice-site gain or loss [73]. By considering an extended genomic sequence context, SpliceAI can identify potential splicing alterations outside canonical donor and acceptor positions, including cryptic splice-site activation. This capability is particularly relevant in the interpretation of rare variants for which the molecular consequence cannot be inferred from genomic position alone.

Several computational approaches have been developed to predict the effects of genomic variants on pre-mRNA splicing, ranging from conventional sequence-based algorithms to machine-learning and deep-learning models. MaxEntScan represents a widely used conventional approach based on maximum-entropy modelling of short sequence motifs at donor and acceptor splice sites [74]. More recent methods exploit machine-learning architectures to capture more complex sequence determinants of splicing. MMSplice adopts a modular framework in which neural-network-based components model different exonic, intronic, and splice-site regions and are subsequently integrated to estimate variant-induced changes in splicing [75]. SQUIRLS instead uses a machine-learning framework to combine multiple sequence- and splicing-related features for the identification of potentially splice-altering variants [76]. Pangolin, similarly to SpliceAI, applies deep-learning models to predict splice-site usage and extends this framework toward modelling splicing across different biological contexts [77]. These methodological differences highlight the transition from motif- and rule-based splice-site scoring toward models capable of learning increasingly complex sequence determinants of splicing. Despite these advances, in silico splice prediction remains subject to important limitations. Predictions can depend on transcript selection and the completeness of transcript annotation, while tissue-specific and developmental-stage-dependent splicing may not be adequately represented by current models [78,79,80]. Moreover, the quantitative modelling of splicing events and the increasing complexity of computational architectures pose important challenges in balancing predictive performance, biological interpretability, and computational feasibility. Deep-intronic variants are particularly challenging because their effects may involve activation of cryptic splice sites or pseudo-exons that are difficult to predict reliably. These limitations are especially relevant in NDDs, where disease-relevant splicing alterations may occur specifically in particular brain regions, cell types, or developmental stages [81]. Experimental validation using RNA from blood or other accessible tissues may therefore be inconclusive when the relevant transcript or splicing event is brain-specific. Consequently, the absence of an abnormal transcript in peripheral tissues should not be considered sufficient to exclude a splice-altering effect in the developing brain.

The increasing integration of splicing predictors into WES interpretation provides an additional layer of evidence for variant prioritization. Nevertheless, computational predictions of aberrant splicing should ideally be supported by RNA-based studies whenever appropriate biological material is available, particularly when the predicted alteration represents a major component of the evidence supporting pathogenicity.

### 3.4. Protein-Truncating Variants and Functional Evidence

Although many computational variant-effect predictors have been developed primarily for missense variation, protein-truncating variants (PTVs), including nonsense and frameshift variants, represent an important class of potentially disease-causing variants in NDDs. Their interpretation cannot rely solely on the predicted introduction of a premature termination codon, as the functional consequence depends on transcript context, exon position, nonsense-mediated decay (NMD), and the underlying disease mechanism [82]. Variants predicted to trigger NMD may result in loss of function through reduced transcript abundance, whereas variants escaping NMD may generate truncated proteins with distinct molecular consequences, including dominant-negative or gain-of-function effects [83]. Consequently, transcript selection and assessment of the expected NMD response are particularly important when interpreting PTVs.

Functional evidence provides an additional layer for resolving variant effects and disease mechanisms. Multiplexed assays of variant effect (MAVEs) enable the parallel experimental characterization of large numbers of variants and can provide calibrated functional evidence for clinical variant interpretation [69,84]. When appropriately validated and calibrated against established pathogenic and benign control variants, functional assay results may contribute to ACMG/AMP evidence within a quantitative Bayesian framework, including estimation of the Odds of Pathogenicity (OddsPath). Importantly, assay measurements should not be directly translated into clinical pathogenicity without considering assay validity, calibration, and the relevance of the measured molecular phenotype to the disease mechanism.

This distinction is particularly relevant to NDD-associated genes, as loss- and gain-of-function mechanisms may result in distinct clinical phenotypes. For example, recent evidence from *ASXL*-related disorders indicates that the functional consequences of truncating variants may depend on their position and susceptibility to nonsense-mediated decay, with NMD-escaping variants potentially resulting in distinct molecular mechanisms [85]. Another practical example of the distinction between loss- and gain-of-function mechanisms concerns genes encoding voltage-gated ion channels. Computational or structural predictions may support candidate variant interpretation but generally cannot establish the direction of the functional effect. Electrophysiological characterization therefore remains critical for demonstrating altered channel activity and distinguishing loss- from gain-of-function mechanisms [86]. Accordingly, AI-assisted prediction, structural modelling, and experimental functional assays should be regarded as complementary rather than interchangeable levels of evidence.

### 3.5. AI-Driven Variant and Candidate Gene Prioritization

While pathogenicity predictors estimate the potential functional or deleterious effect of individual genetic variants, variant prioritization represents a broader analytical task aimed at identifying, among the large number of variants detected by NGS, those most likely to explain the phenotype of a specific patient. This distinction is particularly relevant in WES analysis, where conventional filtering based on allele frequency, predicted molecular consequence, inheritance pattern, and existing gene–disease associations may still retain multiple candidate variants requiring further interpretation. Moreover, pathogenicity alone does not necessarily imply disease relevance, as a potentially deleterious variant may occur in a gene unrelated to the clinical phenotype under investigation.

AI-driven prioritization approaches have therefore been developed to integrate multiple and heterogeneous sources of evidence within a unified analytical framework [39,87]. In addition to variant-level information, these approaches may incorporate gene–disease associations, inheritance models, biological pathways, the literature-derived knowledge, and patient phenotypes represented through standardized HPO terms. This strategy may be particularly advantageous in NDDs, where combinations of partially overlapping phenotypes—such as intellectual disability, autism spectrum disorder, epilepsy, speech and language impairment, hypotonia, and motor dysfunction—can arise from variants in numerous different genes [88,89]. Phenotype-driven prioritization encompasses computational approaches with substantially different methodological foundations. These should not be collectively considered AI or machine-learning methods [87]. Some frameworks primarily rely on ontology-based semantic similarity between patient HPO terms and known disease phenotypes, whereas others integrate knowledge bases, the literature-derived evidence, rule-based inheritance models, variant-level pathogenicity information, or supervised machine-learning algorithms. Despite these methodological differences, they share the objective of reducing the candidate search space by integrating the patient’s phenotypic presentation with genomic or gene-level evidence. In such settings, phenotype-aware AI approaches may improve candidate ranking by identifying genes whose known or predicted disease associations better match the individual clinical profile. Machine-learning and other AI-based strategies can subsequently combine these features to rank candidate variants and genes according to their predicted relevance to the individual clinical presentation [90].

Among the several phenotype-driven prioritization tools, Exomiser represents one of the most established frameworks for joint phenotype–genotype prioritization [91,92]. It combines HPO-based phenotype similarity with variant-level information and inheritance modelling to rank candidate genes and variants, while also exploiting phenotypic information from model organisms to support the prioritization of genes with limited human disease annotation. Genomiser extends this framework to support the prioritization of non-coding variants. Importantly, the performance of these approaches depends on their analytical configuration. Cooperstein et al. (2025) [92], using diagnosed cases from the Undiagnosed Diseases Network, demonstrated that filtering and prioritization parameters can influence the ranking and retention of diagnostic variants and provided practical recommendations for optimizing Exomiser and Genomiser in rare-disease diagnostics. LIRICAL adopts a different strategy based on likelihood-ratio calculations, quantitatively comparing observed patient phenotypes with disease models while incorporating genotype and inheritance information [27]. Phen2Gene primarily performs phenotype-to-gene prioritization by converting patient HPO terms into a ranked list of candidate genes and can therefore be applied even before genomic variants are considered [28]. AMELIE instead emphasizes knowledge extraction from the biomedical literature, matching patient phenotypes and candidate genes with published disease evidence [93]. Xrare more explicitly incorporates machine learning, jointly modelling phenotype similarity and genetic evidence to prioritize potentially causative variants [94]. A comparative overview of the main features of the previously described phenotype-driven prioritization tools is provided in Table 3.

This phenotype-aware approach represents an important transition from isolated variant-effect prediction toward patient-specific genomic interpretation, particularly when multiple plausible candidate variants remain after conventional WES filtering. This distinction becomes particularly important when WES identifies multiple rare candidate variants in the same individual. In such cases, variant-effect scores alone may be insufficient to identify the most plausible molecular explanation of the phenotype. Moreover, most current variant-prioritization frameworks are primarily designed to rank individual variants or genes, an approach that may not fully capture oligogenic contributions to NDDs. Evidence from ASD and other NDD cohorts suggests that combinations of rare variants with individually modest effects may converge on shared biological pathways and collectively contribute to disease susceptibility or phenotypic severity [95,96]. Extending AI-assisted prioritization toward oligogenic models would therefore require evaluating combinations of variants or genes rather than ranking each candidate independently. An example of this approach is ORVAL (Oligogenic Resource for Variant AnaLysis), a platform specifically developed to explore potentially disease-causing oligogenic variant combinations. ORVAL integrates machine-learning-based methods, including the Variant Combination Pathogenicity Predictor (VarCoPP), to evaluate candidate variant combinations across gene pairs and subsequently rank potentially pathogenic gene-pair associations within oligogenic networks [97]. However, such approaches face substantial challenges, including the rapid increase in the number of possible combinations as higher-order interactions are considered, limited statistical power for rare combinations, and the need for replication across populations and ancestral backgrounds. Integrating pathway, protein-interaction, and functional-network information may help constrain this search space and identify biologically plausible combinations, although oligogenic predictions currently require cautious interpretation and independent validation. For example, a trio-based WES in an individual with a complex neurodevelopmental phenotype identified a de novo *USP24* variant together with inherited variants of uncertain significance in *NRXN2* and *LRP2* [31]. Although in silico analyses supported a potential functional impact for all three variants, the de novo occurrence of the *USP24* variant, together with gene-level evidence and phenotype compatibility, supported its prioritization as the principal candidate, while a possible modifying contribution of the inherited variants could not be excluded. More broadly, this example also highlights a limitation of strictly single-variant or single-gene prioritization frameworks, as blended or oligogenic contributions may need to be considered when multiple biologically plausible variants coexist in the same individual. It also illustrates the conceptual distinction between variant-effect prediction, variant prioritization, and candidate-gene prioritization: related, but not interchangeable, components of genomic interpretation.

A further challenge is represented by variants affecting genes for which disease associations are still emerging. Trio-based WES studies have identified de novo variants in *KLF13*, *PSMC5*, and *FAAH2* in individuals with neurodevelopmental or neuropsychiatric phenotypes, while the corresponding gene–disease relationships remain incompletely established [98,99,100]. In such settings, prioritization of emerging candidate genes remains particularly challenging, as limited disease-specific knowledge reduces the effectiveness of approaches relying on established gene–disease associations.

Among emerging approaches, AIMARRVEL provides an example of machine-learning-assisted variant prioritization in which genomic and phenotypic information can be jointly considered to identify candidate variants potentially relevant to the patient’s phenotype [101]. More generally, phenotype-driven prioritization frameworks can exploit semantic similarity between patient HPO profiles and phenotypes associated with known diseases or genes, thereby increasing the relevance of candidate ranking compared with approaches based exclusively on variant-level pathogenicity scores. By integrating phenotypic similarity with genomic evidence, these approaches may therefore help address the extensive locus heterogeneity and clinical overlap that frequently complicate molecular diagnosis in NDDs.

Importantly, AI-driven prioritization should not be considered equivalent to automated variant classification [102]. Rather, prioritization tools help reduce the variant search space and identify plausible candidate genes and variants for subsequent expert evaluation.

### 3.6. Sequence-to-Function Models for Variant Interpretation

Beyond conventional pathogenicity predictors, sequence-to-function (S2F) models represent an emerging class of deep-learning approaches designed to predict how changes in genomic sequence affect molecular and regulatory processes. Rather than assigning a single pathogenicity score, these models link DNA sequence to functional outputs, including gene expression, chromatin accessibility, transcription factor binding, epigenetic modifications, and RNA processing [103]. Thus, S2F models provide an additional level of variant interpretation by potentially linking a genomic alteration to its downstream molecular consequences.

A major advance was represented by Enformer, a deep-learning model combining convolutional neural networks with transformer-based self-attention mechanisms to predict gene expression and chromatin states directly from approximately 200 kb of genomic sequence [104]. By integrating long-range sequence information, Enformer improved the modelling of distal regulatory interactions and enabled prediction of variant effects on gene expression and other regulatory features [104,105].

More recently, AlphaGenome has expanded the S2F paradigm by combining long-range genomic context with high-resolution and multimodal functional predictions [106]. The model processes up to approximately 1 Mb and predicts thousands of functional genomic tracks, including gene expression, chromatin accessibility, histone modifications, transcription factor binding, RNA splicing, and three-dimensional genome organization. By comparing predictions generated for reference and alternative sequences, AlphaGenome can estimate variant-induced changes across multiple molecular outputs, providing mechanistic information that may not be captured by conventional pathogenicity predictors [107]. However, sequence-to-function predictions are inherently dependent on the biological context in which regulatory activity occurs. Sequence-only models may show limited generalization to cell types or conditions not represented during training, indicating that DNA sequence alone may be insufficient to capture context-specific regulatory effects. Recent context-aware approaches have therefore begun to integrate cis-regulatory sequence information with trans-regulatory cellular context. For example, Corgi incorporates a trans-regulatory context vector to support prediction of regulatory activity and variant effects in previously unseen cellular contexts [108]. This limitation is particularly relevant to NDDs, as neurodevelopmental genes can show specific expression patterns across brain regions, cell types, and developmental stages [109]. Accordingly, S2F outputs should be interpreted as context-dependent predictions of molecular consequences rather than context-independent estimates of clinical pathogenicity. When the contribution of rare non-coding variation is of primary interest, WGS rather than WES provides a more appropriate genomic framework.

Emerging AI approaches should also be distinguished according to the biological unit being modelled and the predicted outcome. Sequence-to-function models such as Enformer and AlphaGenome predict molecular genomic readouts directly from DNA sequence and are primarily aimed at estimating the regulatory consequences of sequence variation. By contrast, genomic language or foundation models learn general sequence representations that can subsequently be adapted to variant-level tasks. For example, EVEE uses frozen representations from the Evo 2 genomic foundation model together with a supervised probe to estimate variant effects [110]. These approaches therefore differ in their training objectives, downstream labels, and level of interpretation. Importantly, performance on one variant-assessment task cannot be assumed to transfer across variant classes or regulatory outcomes, making evaluation design, held-out genes or variants, and task-specific validation central to their interpretation.

At the level of rare regulatory variation, category-based association approaches can reduce the analytical burden associated with individually rare regulatory variants by aggregating them according to genomic and functional annotations. For example, CWAS-Plus integrates WGS data with user-defined functional annotations, including cell-type-specific regulatory information, to test categories of rare non-coding variants for association with disease. Its application to ASD incorporated single-nucleus chromatin-accessibility data from the developing human brain, illustrating how cell-type-specific functional information can help contextualize rare regulatory variation [111].

The role of S2F models should be distinguished from that of conventional pathogenicity predictors. Whereas tools such as REVEL or AlphaMissense estimate variant deleteriousness or pathogenicity, AlphaGenome predicts changes in molecular phenotypes resulting from sequence alterations. Its outputs should therefore be interpreted as predictions of molecular consequences rather than direct estimates of clinical pathogenicity [106]. Nevertheless, S2F models may provide complementary information for exonic variants affecting splicing, regulatory sequences captured by exome designs, and variants whose consequences extend beyond amino acid substitution. In fact, S2F models complement variant-level pathogenicity prediction by generating mechanistic hypotheses linking genomic variation to alterations in gene regulation and RNA processing.

### 3.7. Integrating Computational Evidence: Redundancy, Complementarity, and Mechanism Dependence

The evidence reviewed across the preceding sections indicates that the integration of multiple computational approaches does not necessarily increase the strength of variant interpretation. A central distinction should be made between computational redundancy and evidential complementarity. Predictors that share evolutionary annotations, training datasets, or component algorithms may generate concordant outputs without providing independent evidence. Their combined use may support candidate prioritization, but agreement among correlated predictors should not be interpreted as cumulative confirmation of pathogenicity, as illustrated by the concordant and discordant computational predictions observed in the SCN2A examples discussed above.

By contrast, approaches interrogating different dimensions of the genotype–phenotype relationship may provide genuinely complementary information. Variant-effect prediction addresses the predicted molecular consequence of a sequence alteration, whereas phenotype-driven prioritization evaluates its relevance to the clinical presentation. Transcript and splicing analyses address consequences at the RNA level, while experimentally calibrated functional assays can directly investigate specific molecular effects. Structural modelling may provide an additional mechanistic context when a stable altered protein product is expected and the affected residue can be meaningfully interpreted within a reliable structural model. Thus, the potential value of combining computational approaches depends on the biological independence and relevance of the information they provide rather than on the number of tools applied.

This distinction also limits the transferability of conclusions across variant classes and genes. Missense-specific predictors cannot be directly extrapolated to protein-truncating, splice-altering, or regulatory variants. For variants expected to cause loss of function through NMD, transcript context, gene-level constraint, and the established disease mechanism may be more informative than structural modelling. Conversely, structural contextualization may be useful for selected missense variants affecting functional domains, interaction interfaces, or binding environments, whereas regulatory or cellular-context information may be more relevant for dosage-sensitive transcriptional and chromatin regulators. Accordingly, no fixed sequence of AI-assisted analyses can be considered uniformly appropriate across NDD-associated variants.

From a WES-centered perspective, computational tools should therefore be selected according to the question raised by each candidate variant rather than applied as a cumulative sequence of increasingly sophisticated predictions. The principal contribution of an integrated workflow is not to generate a consensus computational classification but to connect variant-level predictions with phenotype, molecular mechanism, and independent evidence while identifying hypotheses that can be experimentally tested.

### 3.8. Limitations and Challenges of AI-Driven Variant Interpretation and Prioritization

Despite their increasing predictive performance, computational and AI-driven tools present several limitations that must be considered during clinical variant interpretation. First, predictive performance can vary substantially according to variant class, gene, molecular mechanism, and the availability of evolutionary information [66,112]. Evolutionary conservation is a major source of information for variant-effect prediction, but the evolutionary history of genes is itself heterogeneous. Gene duplication followed by neofunctionalization or subfunctionalization, exon shuffling, and lineage-specific gene innovation can generate genes, proteins, and protein regions that have experienced different selective trajectories [113]. Accordingly, conservation-based evidence should be interpreted within the broader biological and evolutionary context of the gene rather than as an absolute measure of functional relevance. Supervised models may also inherit biases from their training datasets, including the overrepresentation of extensively studied genes, previously classified variants, specific variant classes, or particular molecular mechanisms. This knowledge bias may be particularly relevant in NDDs, where disease-gene discovery remains incomplete and novel candidate genes continue to emerge from trio-based sequencing studies. Models relying heavily on curated disease databases, the literature-derived information, or previously established gene–phenotype associations may preferentially rank well-characterized NDD genes while under-prioritizing biologically plausible candidates supported by only limited clinical evidence. Furthermore, different predictors frequently incorporate overlapping annotations or training data. Consequently, concordance among multiple variant-effect predictors should not be interpreted as multiple independent lines of evidence. Predictors may share input features, evolutionary conservation metrics, training data, or even outputs from other computational tools. For example, REVEL integrates predictions from SIFT, PolyPhen-2, MutPred, FATHMM, and VEST, together with conservation metrics including GERP++, phyloP, and phastCons, several of which are also incorporated among the annotations used by CADD. Thus, concordant CADD and REVEL scores are partially dependent and should not be treated as independent confirmation of a variant effect. More generally, agreement among predictors may increase confidence in a computational hypothesis, but the number of concordant scores should not be used as a simple measure of evidence strength.

Another important consideration concerns the distinction between deleteriousness, functional impact, and clinical pathogenicity. A variant predicted to alter protein function is not necessarily disease-causing, as clinical pathogenicity additionally depends on gene–disease validity, inheritance pattern, zygosity, penetrance, molecular mechanism, and compatibility with the patient’s phenotype [114]. Consequently, AI-generated variant-effect or deleteriousness scores should not be interpreted in isolation or considered substitutes for established variant-classification frameworks. Rather, population frequency, segregation analysis, evolutionary constraint, functional evidence, phenotype compatibility, and available clinical evidence remain essential components of variant assessment.

Similar considerations apply to AI-driven phenotype-based prioritization. Although HPO-based phenotypic representation provides a standardized framework for computational analysis, its application is not devoid of limitations. The hierarchical organization and semantic redundancy of HPO terms, differences in phenotypic granularity, and incomplete or variable clinical annotation may influence phenotype-similarity estimates and consequently affect candidate gene and variant ranking [115]. These limitations may be particularly relevant in NDDs, where substantial phenotypic overlap and variable expressivity frequently occur across genetically distinct conditions [89]. Emerging language-model-based approaches may help mitigate some of these challenges by supporting automated deep phenotyping from unstructured clinical information. For example, RAG-HPO combines large language models with retrieval-augmented generation to extract phenotypic information from clinical text and map it to standardized HPO terms [116]. In benchmarking against manually assigned HPO annotations, RAG-HPO improved phenotype-extraction performance compared with conventional HPO-matching approaches, while retrieval augmentation substantially reduced unsupported term generation. Such approaches may reduce the burden of manual phenotype annotation and improve the completeness of phenotypic information available for downstream prioritization, although expert review remains necessary before automatically extracted HPO terms are incorporated into genomic interpretation. This limitation may be particularly pronounced in young children, in whom age-dependent manifestations may not yet have emerged, potentially reducing phenotype–gene similarity for the correct molecular diagnosis and favoring genes associated with more completely characterized phenotypes.

A further emerging direction is the transition from binary or general pathogenicity prediction toward phenotype-resolved variant-effect prediction. PheMART, for example, uses deep contrastive learning to associate missense variants with specific clinical phenotypes rather than estimating pathogenicity in isolation, integrating variant representations with protein, interaction, knowledge-graph, and clinical information [117]. Such phenotype-resolved approaches may be particularly relevant to NDDs, in which locus and allelic heterogeneity can result in substantially different clinical manifestations among variants affecting the same gene.

AI-based prediction and prioritization should therefore be considered complementary components of an integrated genomic interpretation process rather than standalone evidence of pathogenicity. Within this framework, computational approaches can substantially reduce the interpretative burden associated with WES by identifying and ranking variants that warrant further clinical, molecular, or experimental investigation. For prioritized coding variants, particularly missense substitutions, AI-based protein structure prediction may provide an additional level of molecular contextualization by mapping affected residues within their three-dimensional environment and supporting the investigation of potential consequences on protein structure and molecular interactions. The opportunities and limitations associated with these structural approaches are discussed in the following section.

## 4. AI and Protein Structural Prediction

### 4.1. Artificial Intelligence in Protein Structure Prediction

The functional consequences of genetic variants are ultimately determined by their effects on biological processes, which may include alterations in protein folding, stability, molecular interactions, or activity [118]. For missense variants in particular, the substitution of a single amino acid may modify local physicochemical properties, disrupt structurally or functionally relevant residues, or interfere with protein–protein and protein–ligand interactions. Consequently, the 3D structural context of a variant can provide valuable complementary information for its biological interpretation [119]. However, experimental determination of protein structures through X-ray crystallography, nuclear magnetic resonance spectroscopy, or cryogenic electron microscopy remains resource-intensive and is not available for a substantial proportion of proteins and protein regions. Notably, experimentally determined protein structures, when available, can be retrieved from the Protein Data Bank (PDB), which represents the primary repository for three-dimensional structural data of biological macromolecules [120].

The application of artificial intelligence to protein structure prediction has substantially changed this scenario. Earlier computational approaches were largely dependent on experimentally determined homologous structures or physics-based simulations, limiting their applicability to proteins lacking suitable templates [15]. Advances in deep learning, together with the increasing availability of protein sequences and experimentally resolved structures, have enabled the development of models capable of learning complex relationships between amino acid sequence, evolutionary constraints, and three-dimensional organization. The emergence of AlphaFold2 represented a major advance in this field, demonstrating the ability of deep neural networks to predict protein structures with high accuracy even in the absence of closely related experimentally determined templates [121].

### 4.2. AI-Based Tools for Protein Structure Prediction

AlphaFold2 integrates information derived from the primary amino acid sequence, multiple sequence alignments (MSAs), and, when available, structural templates through a deep-learning architecture designed to model evolutionary and spatial relationships between residues [121]. The subsequent application of AlphaFold2 at the proteome scale dramatically expanded the structural coverage of the human proteome, providing predicted structures for nearly all human proteins and thereby offering an unprecedented resource for investigating proteins for which experimental structural information is unavailable.

The success of AlphaFold2 was accompanied by the development of alternative deep-learning approaches. RoseTTAFold introduced a three-track neural network architecture in which information at the one-dimensional sequence, two-dimensional distance-map, and three-dimensional coordinate levels is iteratively exchanged during structure prediction [122,123]. Protein language models subsequently provided another strategy for learning structural information directly from large collections of protein sequences. ESMFold, for example, exploits representations learned by a large protein language model to predict three-dimensional structures directly from individual amino acid sequences, reducing its dependence on conventional multiple-sequence-alignment pipelines [124]. These approaches demonstrate how different AI architectures can extract structural constraints from the rapidly expanding amount of protein sequence information. The performance of AlphaFold2, RoseTTAFold2, and ESMFold has been comparatively evaluated in the structural modelling of ion channels using experimentally determined cryo-electron microscopy (cryo-EM) structures as references [125]. As results of this comprehensive comparison, AlphaFold2 showed higher structural accuracy for several of the investigated targets, generally displaying lower Cα-RMSD values than RoseTTAFold2 and ESMFold. However, as emphasized by authors, prediction accuracy varied across individual protein regions and conformational states, highlighting that the relative performance of these approaches may depend on the specific structural context and should not be generalized across all protein classes [125].

A further conceptual advance was introduced by AlphaFold3 [126]. Rather than focusing primarily on individual proteins, AlphaFold3 was designed as a unified deep-learning framework capable of predicting biomolecular complexes containing proteins, DNA, RNA, small-molecule ligands, ions, and modified residues. The model incorporates a substantially redesigned architecture with a diffusion-based structure-generation component and demonstrated improved performance across several categories of biomolecular interactions compared with previous specialized approaches [126]. This extension from protein structure prediction to the modelling of biomolecular interactions is particularly relevant to variant interpretation, since disease-associated amino acid substitutions may exert their effects not only by altering protein folding but also by disrupting interaction interfaces, ligand-binding regions, or macromolecular complexes [127]. AlphaFold3 is accessible through a dedicated, user-friendly web server (https://alphafoldserver.com/) (accessed on 17 August 2026) developed by Google DeepMind, facilitating its application without requiring advanced computational expertise. These access modalities should be distinguished, as differences in computational environments, database versions, input processing, and server-versus-local pipeline settings may affect reproducibility and should therefore be documented when structural predictions are reported. The main characteristics and applications of these approaches are summarized in Table 4.

#### Confidence Assessment and Structural Comparison Metrics

Although current AI-based approaches can generate highly accurate structural models, prediction reliability may vary substantially across proteins and even among different regions of the same protein [15]. Different computational frameworks therefore provide specific confidence metrics that allow the reliability of predicted local and global structural features to be evaluated before their biological interpretation.

**Figure 2:**
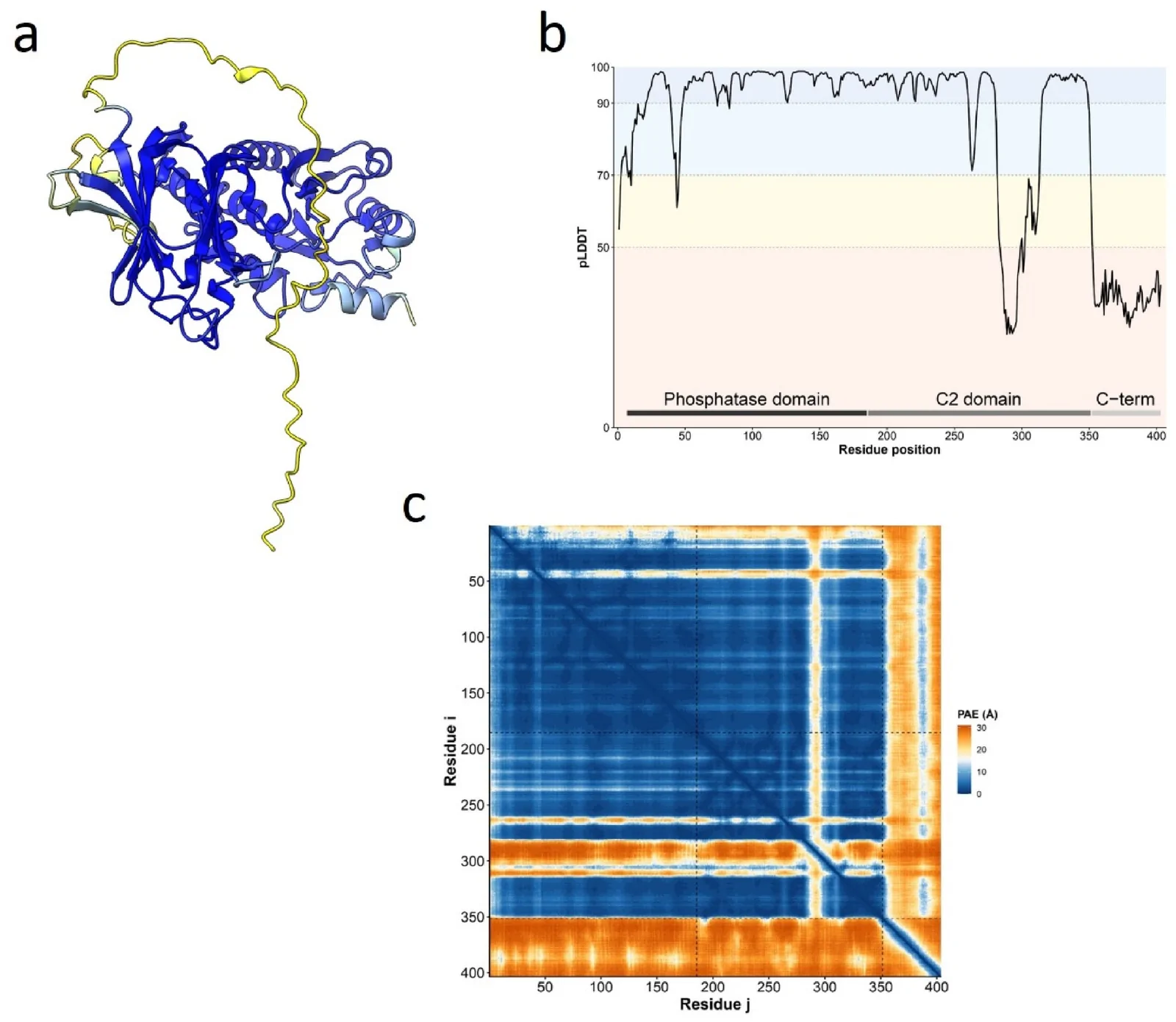
Confidence assessment of the human PTEN protein. (**a**) Three-dimensional AlphaFold model of PTEN obtained from the AlphaFold Protein Structure Database (entry AF-P60484-F1, version v6, accessed on 17 August 2026), visualized and analyzed using UCSF ChimeraX v1.10.1. Residues are colored according to the standard AlphaFold pLDDT confidence scale: dark blue, very high confidence (pLDDT > 90); light blue, confident (70–90); yellow, low confidence (50–70); and orange, very low confidence (<50). (**b**) Residue-level pLDDT profile across the PTEN amino acid sequence, generated using R version 4.5.2 from the confidence values associated with the AlphaFold model. Dashed horizontal lines indicate commonly used confidence thresholds at pLDDT values of 50, 70, and 90, while the major protein regions are indicated along the sequence. (**c**) Predicted Aligned Error (PAE) matrix of PTEN generated in R version 4.5.2 from the PAE data provided with the corresponding AlphaFold prediction. PAE estimates the expected positional error between pairs of residues, with lower values indicating greater confidence in their relative positioning and higher values indicating greater positional uncertainty. Together, pLDDT and PAE provide complementary information regarding local structural confidence and the reliability of the relative positioning of different protein regions. PTEN was selected as a representative NDD-relevant protein because germline PTEN variants are associated with neurodevelopmental phenotypes, including autism spectrum disorder, developmental delay/intellectual disability, and macrocephaly.

Among the most widely used confidence metrics, the predicted Local Distance Difference Test (pLDDT) provides a residue-level estimate of prediction confidence, with higher values indicating greater confidence in the local structural environment [128]. The Predicted Aligned Error (PAE), conversely, estimates the expected positional error between pairs of residues and is particularly informative for assessing confidence in the relative orientation of protein domains or different regions of a predicted structure [129]. AI-based protein structure prediction should be accompanied by an appropriate assessment of model confidence. Different computational frameworks provide specific confidence metrics that estimate the reliability of predicted local and global structural features. Figure 2 shows PTEN as a representative example of confidence assessment in AI-based protein structure prediction, illustrating the three-dimensional distribution and residue-level variation in pLDDT, together with the PAE matrix describing confidence in the relative positioning of residue pairs.

PTEN was selected as a representative example because germline PTEN variants are associated not only with PTEN hamartoma tumor syndrome but also with clinically relevant neurodevelopmental phenotypes, including autism spectrum disorder, developmental delay/intellectual disability, and macrocephaly [130]. Structural contextualization of PTEN variants therefore provides a clinically relevant example of how AI-predicted protein models may complement variant interpretation in NDDs.

Global structural confidence can additionally be evaluated through the predicted Template Modeling score (pTM), whereas predictions involving multiple interacting chains can be assessed using interface-oriented metrics such as the interface-predicted Template Modeling score (ipTM) [131]. The availability and interpretation of these metrics may differ according to the structural prediction framework employed.

Confidence metrics should be clearly distinguished from measures used to compare different protein structures. Structural differences between predicted and/or experimentally determined protein structures can, for example, be quantified using the Root-Mean-Square Deviation (RMSD), which measures the average positional deviation between corresponding atoms following structural alignment [132]. Unlike pLDDT, PAE, and related confidence estimates, RMSD is not an intrinsic measure of prediction reliability but rather a comparative metric calculated between two aligned structures. Low RMSD values generally indicate greater structural similarity, whereas higher values indicate increasing structural divergence; however, RMSD values should always be interpreted in relation to the region and atoms included in the structural alignment.

The distinction between model confidence and structural comparison is particularly important when investigating the potential effects of genetic variants. A high-confidence predicted structure does not necessarily imply that the functional consequence of a variant can be reliably inferred, while structural differences between independently predicted wild-type and mutant models do not by themselves demonstrate protein destabilization or pathogenicity. Confidence metrics and comparative structural measures should therefore be interpreted as complementary information within a broader framework of variant assessment. The principal metrics commonly employed in AI-based protein structure prediction and comparative structural analyses are summarized in Table 5.

### 4.3. Structural Contextualization of Candidate Genetic Variants

Within a WES-based workflow for NDDs variant interpretation, structural modelling can represent a downstream level of investigation following variant filtering and pathogenicity prioritization. Structural contextualization is primarily applicable to missense variants and other alterations that modify protein sequence while retaining a protein product; it is generally less informative for protein-truncating variants expected to result in transcript degradation through NMD. For truncating variants predicted to escape NMD, structural information may occasionally help contextualize the loss of protein domains or interaction regions, although such analyses require caution.

Structural contextualization is applicable only to a subset of prioritized variants, primarily missense variants and other protein-altering variants for which a stable protein product is expected. Its utility is substantially more limited for variants whose primary pathogenic mechanism involves haploinsufficiency, transcript degradation, altered gene dosage, or regulatory dysfunction. Accordingly, structural modelling should not be regarded as a general downstream step following variant prioritization, but rather as one of several mechanism-dependent approaches for the functional contextualization of candidate variants. This distinction is particularly relevant in NDDs, in which many disease-associated genes encode transcriptional and chromatin regulators whose disruption may primarily affect gene dosage and downstream regulatory networks rather than protein folding. For such genes, genomic and regulatory contextualization—including chromatin occupancy, transcriptional targets, and downstream gene-expression networks—may therefore be more informative than structural modelling. For example, Darbandi et al. (2024) [133] showed that five ASD-associated transcriptional regulators (*ARID1B*, *BCL11A*, *FOXP1*, *TBR1*, and *TCF7L2*) converge on shared promoter-proximal loci in open chromatin during cortical development, supporting regulatory convergence as an important functional layer in ASD-associated haploinsufficiency. In the context of NDDs, this may be particularly useful for investigating rare or uncertain variants in genes encoding proteins involved in complex molecular interactions, signalling pathways, or neurodevelopmental processes [134,135]. The position of the altered residue can be evaluated in relation to functional domains, catalytic or binding sites, protein–protein interfaces, secondary structural elements, and the surrounding molecular environment [136]. Structural investigation may therefore help generate hypotheses regarding the potential molecular consequences of amino acid substitutions. Changes in residue size, charge, polarity, or hydrophobicity may potentially modify local interactions, including hydrogen bonds, electrostatic interactions, and hydrophobic contacts. Variants located at interaction interfaces may affect protein–protein associations, whereas substitutions within ligand-binding pockets may influence molecular recognition. The increasing ability of models such as AlphaFold3 to predict complexes containing different classes of biomolecules further expands the possibility of examining candidate variants within a broader molecular context rather than considering the isolated protein alone [137,138]. Notably, the predictive performance of AlphaFold3 has been extensively benchmarked against other computational approaches across different classes of biomolecular interactions, demonstrating its strong capabilities in modelling complex biological systems.

However, an important distinction should be made between structural prediction and variant-effect prediction. AlphaFold and related tools were primarily developed to predict likely protein structures rather than to quantify the energetic or pathogenic consequences of amino acid substitutions. Direct comparison of independently predicted wild-type and mutant structures, including differences in RMSD, hydrogen bonding, or local geometry, should therefore be interpreted cautiously [139]. Importantly, structural differences, including changes in RMSD, should not be interpreted as direct evidence of pathogenicity. Such differences may reflect model uncertainty or alternative predicted conformations rather than a genuine variant-induced structural effect and therefore require independent functional or experimental validation. Structural models are consequently most informative when used to contextualize a candidate variant and formulate mechanistic hypotheses that can subsequently be investigated using dedicated stability calculations, molecular dynamics, biochemical assays, or other experimental approaches.

**Figure 3:**
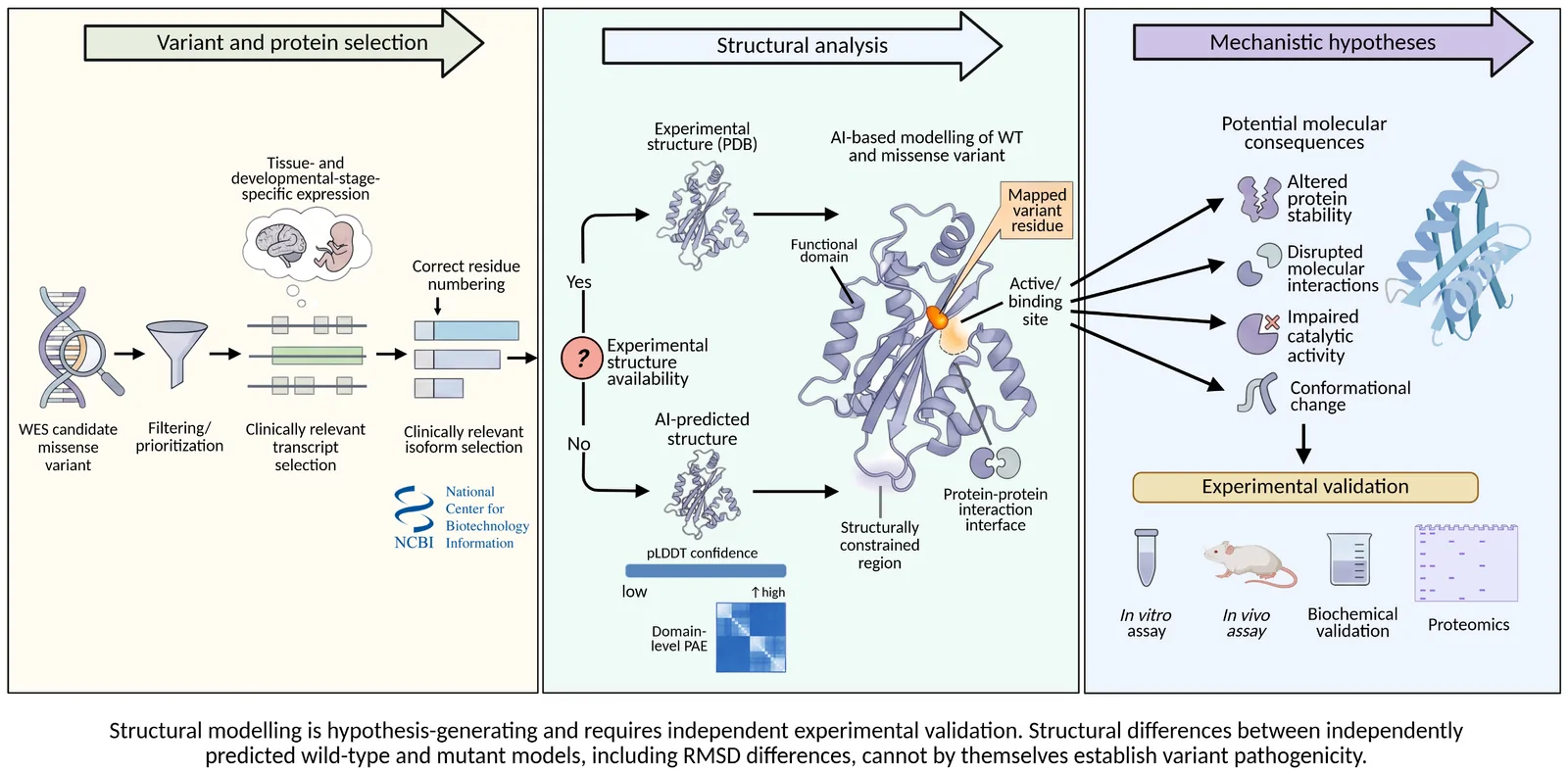
Conceptual workflow illustrating the potential integration of structural contextualization into the interpretation of candidate missense variants identified by WES in NDDs. Following variant filtering and prioritization, the clinically relevant transcript and corresponding protein isoform are selected, considering tissue- and developmental-stage-specific expression and correct residue numbering. The availability of experimentally determined structures (PDB) is then assessed, while AI-based structural modelling can provide complementary models of the wild-type and missense variant. Predicted structures should be evaluated using confidence metrics, including residue-level pLDDT and domain-level PAE, before mapping the affected residue and assessing its localization within functional domains, active or binding sites, protein–protein interaction interfaces, or structurally constrained regions. Structural observations can subsequently support mechanistic hypotheses regarding potential effects on protein stability, molecular interactions, catalytic activity, or conformation, which require experimental validation through appropriate in vitro, in vivo, biochemical, or proteomic approaches. Importantly, structural modelling should be considered hypothesis-generating, and differences between independently predicted wild-type and mutant structures, including RMSD differences, cannot independently establish variant pathogenicity. The workflow is intended as a conceptual representation of complementary interpretive steps rather than as a validated diagnostic pipeline.

In this framework, AI-based structural prediction complements rather than replaces pathogenicity prediction. Tools such as REVEL, AlphaMissense, PrimateAI-3D, or EVE address the probability or likelihood that a variant may have a deleterious or disease-associated effect, whereas structural models can provide information regarding the molecular environment in which the affected residue operates. Integrating these complementary levels of evidence may therefore provide a more comprehensive interpretation of prioritized WES variants. In the context of NDD variant interpretation, structural modelling may be integrated into a stepwise workflow following variant prioritization. First, the clinically relevant transcript and corresponding protein isoform should be selected, considering disease-associated transcripts and tissue- or developmental-stage-specific expression when available. The availability of experimentally determined structures should then be assessed before relying on predicted models. When AI-derived structures are used, global and local confidence metrics should be examined, including residue-level pLDDT and domain-level PAE. The variant can subsequently be mapped onto the three-dimensional structure to determine its localization within a functional domain, catalytic or binding site, protein–protein interaction interface, or structurally constrained region. This structural context may support mechanistic hypotheses regarding altered protein stability, domain organization, or molecular interactions, which can subsequently guide experimental validation. A proposed workflow for integrating these steps into WES-based NDD variant interpretation, from transcript and protein isoform selection to structural contextualization and experimental validation, is summarized in Figure 3.

### 4.4. Current Limitations of AI-Based Structural Prediction

Despite their remarkable performance, AI-based structural prediction models present several limitations that are particularly important when applied to human genetic variation. Prediction accuracy is not uniform across proteins, particularly in intrinsically disordered or highly flexible regions, for which a single stable three-dimensional conformation may not adequately represent their biological state [140]. Interpretation may also be complicated by alternative protein isoforms, membrane proteins, post-translational modifications, interaction partners, and biologically relevant conformational states. Moreover, isolated protein models may not reproduce the native environment of multimeric complexes or membrane-associated systems. Consequently, predicted structural alterations should be interpreted in the context of model confidence, protein function, and available experimental evidence.

Confidence metrics such as pLDDT and PAE should therefore always be considered before drawing conclusions from a predicted structure. In particular, protein regions characterized by limited evolutionary information, high conformational flexibility, or intrinsic disorder may exhibit lower prediction confidence. In such cases, low-confidence scores indicate greater uncertainty in the predicted structural organization and warrant caution when interpreting the potential structural consequences of genetic variants. This issue may be particularly relevant in the context of NDDs, where several disease-associated or candidate genes remain functionally and structurally poorly characterized and experimentally determined protein structures may not be available [141]. In these cases, structural investigation necessarily relies more heavily on computationally predicted models. Importantly, the absence of an experimentally determined structure does not itself imply low prediction confidence, as AI-based approaches may generate highly confident predictions even for proteins lacking experimental structural data. Nevertheless, some poorly characterized proteins or protein regions may also be affected by limited evolutionary information, intrinsic disorder, or conformational flexibility, resulting in low-confidence predictions [142].

When a candidate missense variant falls within such a region, structural comparisons between wild-type and mutant models should be interpreted with particular caution. Apparent variant-induced changes in local conformation, residue interactions, or global structural parameters may partly reflect uncertainty in the underlying structural model rather than a genuine molecular consequence of the amino acid substitution [140]. Accordingly, confidence in the reference structural model, particularly within the region surrounding the affected residue, should represent an essential consideration when interpreting variant-induced structural differences.

Second, proteins are dynamic molecular entities that frequently adopt multiple conformational states. Most structure-prediction approaches provide one or a limited number of structural hypotheses and may consequently fail to capture conformational ensembles, transient states, allosteric transitions, and other dynamic properties that are fundamental to protein function [143]. This limitation becomes particularly relevant when investigating disease-associated variants whose effects may arise from changes in protein dynamics rather than major alterations in the equilibrium structure. Recent sequence-based models have begun to extend structural prediction beyond static conformations by predicting dynamic residue contacts, contact occupancy, and aspects of conformational behaviour directly from protein sequence [144]. However, the integration of such dynamic predictions into routine variant interpretation and clinical genomic workflows remains insufficiently established and requires further validation.

Third, high structural confidence does not imply that the predicted biological effect of a variant is equally reliable [145]. AI models may reproduce highly similar structures for wild-type and mutant sequences even when experimental evidence demonstrates substantial differences in protein stability or function. Conversely, apparent differences between independently generated predictions may reflect model uncertainty rather than genuine variant-induced conformational changes. Therefore, structural similarity or divergence between wild-type and mutant models should not be directly interpreted as evidence for or against variant pathogenicity. Protein structure predictions should instead be regarded as a means of providing molecular context and generating testable mechanistic hypotheses. An additional limitation concerns the representation of cofactors, metal ions, and ligands, which can be essential for protein stability, catalytic activity, and molecular interactions but may be absent or inaccurately modelled in predicted structures, thereby limiting the interpretation of variants affecting their binding or coordination environments [146].

Finally, computational structural modelling does not replace experimental validation. Whenever feasible, predicted effects should be evaluated using complementary approaches such as protein stability measurements, interaction assays, expression studies, cellular models, or other functional experiments relevant to the proposed disease mechanism [140]. Within neurogenomic diagnostics, the principal value of AI-based structural prediction therefore lies in providing an additional layer of molecular contextualization for variants already prioritized through genomic, computational, phenotypic, and clinical evidence.

## 5. Challenges and Future Perspectives

Future developments in AI-assisted neurogenomics are expected to move beyond individual prediction tasks toward increasingly integrated and multimodal frameworks. The progressive extension from WES toward genome-wide and multi-omic approaches is likely to broaden both the spectrum of detectable variation and the molecular dimensions available for AI-assisted interpretation. Integration of coding and non-coding variation, structural variation, transcriptomic and epigenomic information, chromatin organization, molecular networks, and protein-level consequences may ultimately provide a more comprehensive representation of the molecular architecture underlying genetically heterogeneous disorders [147,148,149]. Such a transition will require computational frameworks capable of integrating evidence across different levels of genome organization rather than treating individual sequence variants as isolated events. This transition may be particularly valuable in NDDs, where extensive genetic heterogeneity, phenotypic overlap, and variable expressivity frequently complicate molecular diagnosis.

Nevertheless, the clinical implementation of these approaches will require rigorous external validation, improved model interpretability, and standardized benchmarking. Greater representation of diverse populations in genomic reference datasets and AI model development will also be important to improve allele-frequency filtering, calibration, and the reliability of variant interpretation across ancestral backgrounds. Importantly, recent large-scale sequencing of Latin American individuals with ASD suggests that the contribution of deleterious coding variation to autism is largely shared across ancestries, indicating that the need for greater population diversity primarily concerns the representativeness of genomic reference data and the calibration of interpretive models rather than fundamentally ancestry-specific disease biology [150]. AI should complement rather than replace expert genomic interpretation, with computational predictions integrated alongside clinical, segregation, functional, and experimental evidence [43,44]. Future AI-assisted prioritization frameworks may also need to move beyond conventional single-variant and single-gene ranking toward models capable of evaluating potentially interacting rare variants and their convergence on shared neurodevelopmental pathways. Such approaches may provide a more appropriate framework for investigating oligogenic and modifier effects in genetically complex NDDs.

A further emerging direction is the integration of cellular and developmental context as an additional layer of genomic interpretation. Beyond estimating variant-level molecular effects or structurally contextualizing protein-altering variants, understanding where and when a candidate gene acts may be particularly important in NDDs, in which gene function and disease mechanisms can depend on specific neural cell types and developmental states. At a different level of biological organization, emerging models aim to predict gene-perturbation responses within specific cellular contexts rather than variant effects directly. Preliminary approaches such as NOCAP integrate neural-organoid atlases, perturbation-informed modelling, and family-based genomic data to derive functional priors for autism gene prioritization [151], whereas AnnFlux uses object-conditioned neural stochastic differential equations to model distributions and trajectories of cellular responses to perturbations [152]. These approaches may provide functional context for candidate genes, but their outputs should not be conflated with evidence of variant causality. In particular, a predicted cellular response does not establish that a specific variant is pathogenic, and a modelled perturbation trajectory does not represent a directly observed developmental history. Their relevance to NDD variant interpretation will therefore depend on validation across independent perturbations, genes, cellular contexts, and developmental states. More broadly, these emerging method families address different biological levels and should therefore be evaluated according to the specific task for which they were developed. Performance on variant-effect benchmarks does not necessarily establish performance for regulatory-effect prediction, while accurate modelling of gene-perturbation responses does not establish variant pathogenicity or clinical utility in NDD cohorts. Evaluation across held-out genes, variant classes, cellular contexts, and independent datasets will therefore be essential for assessing their transferability. Future computational approaches may further incorporate the temporal dynamics of cellular responses to genetic perturbation rather than relying exclusively on static or endpoint measurements. Such context-dependent frameworks could complement variant-effect prediction and structural modelling by providing an additional functional layer; however, their outputs should be regarded as hypothesis-generating functional priors requiring independent experimental validation. Finally, the clinical implementation of AI-assisted genomic interpretation raises important methodological, ethical, and practical challenges. Population underrepresentation in genomic databases may affect allele-frequency filtering, model calibration, and the interpretation of rare variants, potentially reducing performance across different ancestral backgrounds. Additional concerns include genomic-data privacy, particularly when proprietary or cloud-based models are used, limited model transparency, and reproducibility across different software or model versions [153,154]. Moreover, automation bias may lead to excessive reliance on computational recommendations, emphasizing the need for qualified professionals to critically review AI-generated outputs and integrate them with clinical, genetic, and experimental evidence. Variant interpretation should be considered a dynamic process, as continuous updates to clinical databases, gene–disease associations, classification criteria, and computational models may modify previously generated interpretations, supporting the need for periodic reinterpretation of unresolved or uncertain cases. Beyond diagnostic interpretation, improved characterization of the molecular mechanisms underlying genetic disorders may also have future implications for therapeutic stratification. Distinguishing loss-of-function, gain-of-function, splice-altering, or structurally disruptive mechanisms may become increasingly relevant as gene replacement, gene silencing, RNA-based therapies, and genome-editing approaches are developed for rare genetic disorders [155,156]. Computational approaches may contribute to generating hypotheses regarding these mechanisms; however, mechanism-specific experimental validation remains essential, particularly when the direction of the functional effect has clinical implications. In this perspective, AI-assisted variant interpretation could contribute not only to candidate prioritization but also to the mechanistic characterization that may inform increasingly personalized therapeutic strategies. The growing relevance of genotype- and mechanism-oriented therapeutic approaches is already evident in other rare genetic diseases, in which the specific molecular defect can influence the choice among different molecular therapeutic strategies [157].

## 6. Conclusions

The principal conclusion emerging from this narrative review is that the value of AI-assisted WES interpretation does not derive from accumulating concordant computational predictions but from integrating complementary evidence selected according to variant class and expected disease mechanism. Predictors sharing annotations, training data, or evolutionary information may be useful for candidate prioritization but should not be considered independent evidence. Conversely, phenotype–gene concordance, transcript consequences, inheritance and segregation, experimentally validated functional evidence, and mechanism-appropriate structural or regulatory contextualization can interrogate distinct dimensions of a candidate variant.

This distinction is particularly relevant in NDDs because disease-associated variation encompasses heterogeneous molecular mechanisms, including altered protein function, aberrant splicing, haploinsufficiency, regulatory dysfunction, and potentially oligogenic contributions. No single computational workflow is therefore uniformly applicable across genes and variant classes. The conceptual framework discussed in this review is intended to guide the selection and critical interpretation of existing AI-assisted tools rather than to constitute a validated diagnostic pipeline. Their clinical value ultimately depends on calibrated performance, reproducibility, biological relevance, and integration with independent clinical, genetic, and functional evidence.

## Figures and Tables

**Table 1:** Computational and AI-based tools for variant-effect prediction. Tools were selected as representative examples of machine-learning and deep-learning approaches with distinct methodological frameworks, rather than to provide an exhaustive ranking of available predictors.

Tool	Model	Main Features	Score and Interpretation	Principal Limitations
CADD [49]	Supervised machine-learning integrative model	Applicable to SNVs and small indels across coding and non-coding regions. Integrates conservation, regulatory, and functional annotations; trained to distinguish simulated variants from variants that survived human evolutionary selection	CADD raw score and PHRED-scaled score. Higher scores indicate greater predicted deleteriousness; PHRED ≥ 20 corresponds approximately to the top 1% of variants ranked by CADD	Predicts deleteriousness rather than clinical pathogenicity; dependent on incorporated annotations and training data.
REVEL [51]	Supervised ensemble machine learning (Random Forest)	Designed for rare missense variants. Integrates 18 scores from 13 individual functional and conservation predictors, including SIFT, PolyPhen-2, MutPred, FATHMM, VEST, GERP++, phyloP and phastCons	0–1. Higher values indicate a greater predicted probability/propensity of pathogenicity; the original score represents the proportion of trees classifying the variant as pathogenic.	Missense-specific; correlated component predictors
PrimateAI [52]	Deep residual neural network; semi-supervised learning strategy	Designed for missense variants. Uses amino-acid sequence context, evolutionary conservation, and protein structural features; training exploits common variants observed in humans and non-human primates	0–1. Values closer to 0 indicate lower predicted pathogenicity, whereas values closer to 1 indicate higher predicted pathogenicity.	Dependence on evolutionary representation; missense-specific
PrimateAI-3D [60]	Deep learning incorporating 3D protein information	Designed for missense variants. Extends the PrimateAI concept by incorporating three-dimensional protein structural context together with evolutionary information	Continuous pathogenicity score; higher values indicate greater predicted deleteriousness/pathogenicity	Structural/evolutionary context dependent
EVE [57]	Unsupervised deep generative model—Bayesian variational autoencoder (VAE)	Designed for missense variants in proteins with sufficient evolutionary sequence information. Learns evolutionary constraints from multiple sequence alignments without requiring clinical pathogenic/benign labels for model training	EVE score/evolutionary-index-derived classification. Higher pathogenicity-oriented scores indicate greater compatibility with the pathogenic distribution; also provides class assignment and uncertainty	Requires informative MSAs; coverage varies among proteins
AlphaMissense [53]	Deep-learning model derived from AlphaFold architecture	Designed for missense variants. Combines evolutionary information and protein sequence/structural context; adapted from AlphaFold-based representations for missense variant effect prediction	0–1 pathogenicity score. Higher values indicate greater predicted pathogenicity; scores are additionally categorized into likely benign, ambiguous, and likely pathogenic ranges	Missense-specific; predictions do not constitute clinical variant classification

Notes: Selected tools differ in computational architecture, training strategy, input features, and score calibration. CADD and REVEL are supervised machine-learning approaches; PrimateAI, PrimateAI-3D, and AlphaMissense use deep-learning frameworks incorporating evolutionary and/or structural information. EVE is an unsupervised generative model based on evolutionary constraints. Higher scores generally indicate greater predicted deleteriousness or pathogenicity, although score definition and interpretation are tool-specific. Reported thresholds, where applicable, refer to those proposed in the original tool publications or subsequent calibration studies and should not be interpreted as equivalent to formal clinical variant classification. Accessibility differs among predictors: scores and implementations may be available through dedicated web interfaces, software repositories, or downloadable precomputed datasets. Precomputed scores for several commonly used predictors are also available through integrated annotation resources such as dbNSFP. Tool versions, database releases, calibration sources, and access conditions should therefore be considered and reported when these predictors are applied to variant interpretation.

**Table 2:** Comparison of computational predictions for two *SCN2A* missense variants with contrasting prediction patterns: NM_001040143.1:c.643G>A, p.(Ala215Thr) and NM_001040142.2:c.1399G>A, p.(Ala467Thr).

*SCN2A* Variant	Predictor	Source/Version	Score	Prediction
p.(Ala215Thr)	CADD	ProtVar; CADD v1.7	29.5	High predicted deleteriousness
	REVEL	dbNSFP v5.4	0.866	Deleterious
	AlphaMissense	ProtVar Data release 2.1	0.88	Likely pathogenic
p.(Ala467Thr)	CADD	ProtVar; CADD v1.7	23.5	Potential deleteriousness
	REVEL	dbNSFP v5.4	0.269	Benign-supporting
	AlphaMissense	ProtVar Data release 2.1	0.08	Likely benign

Notes: Scores are reported as illustrative examples of variant-effect prediction and should not be directly compared across models. These examples do not constitute an independent benchmarking analysis, and concordant predictions should not be considered independent evidence because predictors may share underlying features or annotations. CADD and AlphaMissense scores were retrieved through ProtVar, whereas REVEL scores were obtained from dbNSFP. Database resources were accessed on 17 August 2026. ACMG/AMP criteria were retrieved using the VarSome automated classification framework (v13.18.2.1; accessed on 30 September 2026) and were considered together with the available clinical, genetic, and gene-specific evidence. ACMG/AMP criteria reported by VarSome were as follows: PM1, variant located in a mutational hot spot or critical and well-established functional domain; PM2, absent or extremely rare in population databases; PM5, novel missense change at an amino acid residue where a different pathogenic missense change has been established; PP2, a missense variant in a gene with a low rate of benign missense variation and in which missense variants represent a common disease mechanism; PP3, computational evidence supporting a deleterious effect; PP5, pathogenic classification reported by a reputable source without independently available evidence; BS1, allele frequency greater than expected for the disorder; BS2, observation in healthy individuals inconsistent with full penetrance at an early age; BP4, computational evidence supporting no impact on gene or gene product; and BP6, benign classification reported by a reputable source without independently available evidence. PP5 and BP6 are reported here as returned by the VarSome automated framework; these criteria should be interpreted cautiously in light of current ClinGen recommendations and were not considered independent evidence in the discussion of these variants.

**Table 3:** Representative phenotype-driven prioritization tools and their main methodological characteristics. HPO, Human Phenotype Ontology; VCF, Variant Call Format. Tool accessibility and implementation characteristics may vary according to software version and platform.

Tool	Approach	Input → Output	Main Features/Limitations
Exomiser	Ontology-/knowledge-based phenotype–genotype integration	VCF + HPO → genes/variants	Integrates phenotype, variant, and model-organism evidence; dependent on phenotype quality and knowledge bases
Genomiser	Ontology-/knowledge-based phenotype–genotype integration	Genomic variants + HPO → genes/variants	Extends phenotype-driven prioritization to coding and non-coding variants; performance depends on phenotype quality, variant annotations, and analytical configuration
LIRICAL	Likelihood-based phenotype–genotype modelling	HPO + genomic data → diagnoses	Probabilistic and interpretable; dependent on phenotype and disease annotations
Phen2Gene	HPO-based prioritization	HPO → genes	Rapid phenotype-to-gene ranking; no direct variant prioritization
AMELIE	Literature-/knowledge-based prioritization	HPO + genomic data → candidate diagnoses	Integrates literature evidence; dependent on available gene–disease knowledge
Xrare	ML + phenotype/genetic evidence	Variants + phenotype → genes/variants	ML-based integration; dependent on training data and annotations
AIMARRVEL	ML-based phenotype–genotype prioritization	VCF + HPO → genes/variants	Integrates genomic and phenotypic evidence; dependent on phenotype completeness and disease knowledge

**Table 4:** AI-based tools for protein structure prediction and biomolecular modelling.

Tool	AI Approach	Prediction	Application	Principal Limitations
AlphaFold2	Deep neural network	Protein 3D structure	Residue/domain structural context	Limited representation of conformational dynamics; confidence does not establish variant pathogenicity
AlphaFold3	Deep learning + diffusion	Biomolecular complexes	Protein–protein, protein–DNA/RNA, ligand interactions	Computationally demanding for local execution; server/local settings may affect reproducibility; limited representation of conformational dynamics.
RoseTTAFold	Three-track neural network	Protein structure	Protein folding/structural context	Performance may depend on evolutionary information; limitations vary across implementations
ESMFold	Protein language model	Protein structure	Rapid sequence-to-structure prediction	Rapid and MSA-free, but prediction accuracy may be lower for difficult targets; limited complex modelling

Notes: MSA, multiple sequence alignment; AI, artificial intelligence.

**Table 5:** Metrics for confidence assessment and structural comparison.

Metric	Category	Level	Output	Interpretation
pLDDT	Model confidence	Local/residue	0–100	Higher = greater local confidence
PAE	Model confidence	Residue pairs/domains	Å	Lower = greater confidence in relative positioning
pTM	Model confidence	Global	0–1	Higher = greater global confidence
ipTM	Interface confidence	Protein complex/interface	0–1	Higher = greater interface confidence
RMSD	Structural comparison	Global or selected region	Å	Lower = greater structural similarity

Notes: pLDDT, PAE, pTM, and ipTM provide model-specific estimates of prediction confidence at different structural levels, whereas RMSD is a structural comparison metric that quantifies differences between aligned structures and is not an intrinsic measure of prediction confidence. Å, ångström; pLDDT, predicted Local Distance Difference Test; PAE, Predicted Aligned Error; pTM, predicted Template Modeling score; ipTM, interface predicted Template Modeling score; RMSD, Root Mean Square Deviation.

## Data Availability

No primary datasets were generated or analyzed in this review. The computational data used for the illustrative SCN2A examples in Table 2 were retrieved from publicly available resources. CADD and AlphaMissense scores were obtained through ProtVar, and REVEL scores from dbNSFP (accessed on 17 August 2026); population-frequency information was retrieved from gnomAD v4.1, and automated ACMG/AMP classifications from VarSome (accessed on 30 September 2026). The AlphaFold model used in Figure 2 was obtained from the AlphaFold Protein Structure Database.

## Abbreviations

The following abbreviations are used in this manuscript:

ACMG	American College of Medical Genetics and Genomics
AI	Artificial intelligence
ASD	Autism spectrum disorder
CADD	Combined Annotation Dependent Depletion
CNV	Copy number variant
DL	Deep learning
DSM-5	Diagnostic and Statistical Manual of Mental Disorders, Fifth Edition
EVE	Evolutionary Model of Variant Effect
HPO	Human Phenotype Ontology
ID	Intellectual disability
ipTM	Interface predicted template modeling score
MAVE	Multiplexed assays of variant effect
ML	Machine learning
MSA	Multiple sequence alignments
NDD	Neurodevelopmental disorder
NGS	Next-generation sequencing
NMD	Nonsense-mediated decay
ORVAL	Oligogenic Resource for Variant AnaLysis
PAE	Predicted Aligned Error
pLDDT	Predicted Local Distance Difference Test
pTM	Predicted Template Modeling score
S2F	Sequence-to-function
REVEL	Rare Exome Variant Ensemble Learner
RMSD	Root-Mean-Square Deviation
WES	Whole exome sequencing
